# Short-acting β_2_-agonist prescription patterns in patients with asthma in Turkey: results from SABINA III

**DOI:** 10.1186/s12890-022-02008-9

**Published:** 2022-06-02

**Authors:** Arzu Yorgancıoğlu, Kurtuluş Aksu, Sibel Atış Naycı, Dane Ediger, Dilşad Mungan, Umut Gül, Maarten J. H. I. Beekman, SABINA Turkey Study Group, Deniz Kızılırmak, Nejat Altıntaş, İsmet Bulut, Tülin Çağatay, Bilun Gemicioğlu, Özgür İnce, Kıvılcım Oğuzülgen, Füsun Kalpaklıoğlu, Ayşe Baççıoğlu, Funda Aksu, Murat Altuntaş, Ferda Öner Erkekol, Gül Karakaya, Ali Fuat Kalyoncu, Ebru Damadoğlu, İsmail Hanta, Ersoy Altunok, Adviye Özer, Demet Polat Yuluğ, Gazi Gülbaş, Mecit Süerdem, Burcu Yormaz, Emel Ceylan, Duygu Erge, Aykut Çilli, Berat Celil Doğan, Fuat Erel, Can Sevinç, Ceyda Anar, Gülseren Pekbak, Müge Erbay

**Affiliations:** 1 Department of Pulmonology, Faculty of Medicine, Manisa Celal Bayar University, Manisa, Turkey; 2 Division of Immunology and Allergy, Department of Chest Diseases, University of Health Sciences Atatürk Chest Diseases and Chest Surgery Education and Research Hospital, Ankara, Turkey; 3 Department of Pulmonology, Faculty of Medicine, Mersin University, Mersin, Turkey; 4 Division of Allergy and Immunology, Department of Pulmonology, Faculty of Medicine, Bursa Uludağ University, Bursa, Turkey; 5 Department of Pulmonology, Division of Allergy and Immunology, Faculty of Medicine, Ankara University, Ankara, Turkey; 6 AstraZeneca, Istanbul, Turkey; 7 AstraZeneca, The Hague, The Netherlands; 8 Dokuz Eylül Üniversitesi Tıp Fakültesi, İzmir, Turkey

**Keywords:** Asthma, Exacerbations, Turkey, Prescriptions, Short-acting β_2_-agonists

## Abstract

**Background:**

Over-reliance on short-acting β_2_-agonists (SABAs) is associated with poor asthma outcomes. However, the extent of SABA use in Turkey is unclear owing to a lack of comprehensive healthcare databases. Here, we describe the demographics, disease characteristics and treatment patterns from the Turkish cohort of the SABA use IN Asthma (SABINA) III study.

**Methods:**

This observational, cross-sectional study included patients aged ≥ 12 years with asthma from 24 centres across Turkey. Data on sociodemographics, disease characteristics and asthma treatments were collected using electronic case report forms. Patients were classified by investigator-defined asthma severity (guided by the 2017 Global Initiative for Asthma [GINA]) and practice type (primary/specialist care). The primary objective was to describe SABA prescription patterns in the 12 months prior to the study visit.

**Results:**

Overall, 579 patients were included (mean age [standard deviation; SD]: 47.4 [16.1] years; 74.3% female), all of whom were treated by specialists. Most patients had moderate-to-severe asthma (82.7%, GINA steps 3–5), were overweight or obese (70.5%), had high school or university/post-graduate education (51.8%) and reported fully reimbursed healthcare (97.1%). The mean (SD) asthma duration was 12.0 (9.9) years. Asthma was partly controlled/uncontrolled in 56.3% of patients, and 46.5% experienced ≥ 1 severe exacerbation in the preceding 12 months. Overall, 23.9% of patients were prescribed ≥ 3 SABA canisters in the previous 12 months (considered over-prescription); 42.9% received no SABA prescriptions. As few patients had mild asthma, only 5.7% were prescribed SABA monotherapy. Therefore, most patients (61.5%) were prescribed SABA in addition to maintenance therapy, with 42.8% receiving ≥ 3 SABA canisters in the previous 12 months. Inhaled corticosteroids (ICS), ICS + a long-acting β-agonist fixed-dose combination and oral corticosteroids were prescribed to 14.5%, 88.3% and 28.5% of all patients, respectively. Additionally, 10.2% of patients purchased SABA over the counter, of whom 27.1% purchased ≥ 3 canisters in the preceding 12 months.

**Conclusions:**

Despite all patients being treated by specialists and most receiving fully reimbursed healthcare, nearly a quarter of patients received prescriptions for ≥ 3 SABA canisters in the previous 12 months. This highlights a public health concern and emphasizes the need to align clinical practices with the latest evidence-based recommendations.

**Supplementary Information:**

The online version contains supplementary material available at 10.1186/s12890-022-02008-9.

## Background

Asthma, a chronic inflammatory disease of the airways affecting approximately 339 million patients worldwide [1], is associated with a significant social and economic burden [2]. Although information on the epidemiology of asthma in the Middle East, including Turkey, remains limited, results from the SNAPSHOT observational study conducted in five countries in the Gulf cluster reported that the adjusted prevalence of asthma in the general adult population aged > 18 years was 4.4% in Turkey [3]. Despite the availability of updated international and national guidelines and an effective array of medications, poor adherence to treatment guidelines and suboptimal asthma control continue to be reported globally, including in Turkey [4–6]. For example, results from the 2006 Asthma Insights and Reality in Turkey (AIRET) study in 400 patients with asthma surveyed across 15 cities indicated that guideline-based asthma control was achieved in only 1.3% of participants. Moreover, daily use of anti-inflammatory therapy, including inhaled corticosteroids (ICS), was low in those with persistent asthma [6]. Unsurprisingly, according to the 2018 Global Asthma Report, Turkey reported the highest rate of hospital admissions for asthma (all ages) among 30 European countries between 2011 and 2015 [1]. In addition, results from two cost-of-illness studies in Turkey highlighted that the management of asthma exerts a significant economic burden on the country in terms of annual direct medical costs, predominantly drug treatments and hospitalisations [7, 8].

Adherence to evidence-based treatment guidelines is essential to achieve improved clinical outcomes for patients with asthma [9]. However, many patients rely on short-acting β_2_-agonists (SABAs) for rapid symptom relief, even though they have no inherent anti-inflammatory activity and SABA use without concomitant ICS may be pro-inflammatory [10, 11]. Since SABA overuse (≥ 3 canisters/year) is associated with poor disease control and an increased risk of exacerbations, hospitalisations and mortality [12–16], the Global Initiative for Asthma now recommends as-needed low-dose ICS-formoterol as the preferred reliever for adults and adolescents with mild asthma and for those with moderate-to-severe asthma who are prescribed ICS-formoterol maintenance therapy [9].

An examination of the prevalence of SABA use is required to help clinicians and healthcare policymakers fully understand the extent of SABA overuse and ensure that treatment practices align with the latest evidence-based treatment recommendations, particularly in light of updated GINA treatment recommendations [9]. However, to date, there is a paucity of data on trends in asthma medication use in Turkey, primarily due to a lack of comprehensive healthcare databases. Accordingly, Turkey was included in the SABA use IN Asthma (SABINA) III pillar of the SABINA Programme to describe the global extent of SABA use and its clinical consequences through a series of real-world observational studies applying a harmonised approach to data collection, evaluation and interpretation [17]. SABINA III, conducted in 23 countries across the Asia–Pacific, Africa, the Middle East, Latin America and in Russia, used electronic case report forms (eCRFs) to overcome the lack of robust national healthcare databases in many of the participating countries [18]. Here, we report on results from SABINA Turkey to provide real-world evidence on asthma management practices in this country.

## Methods

### Study design

Detailed methodology for SABINA III has been published previously [18]. In brief, this was an observational, cross-sectional study conducted at 24 centres in Turkey, with patient recruitment from September 2019 to January 2020. The study sites were selected using purposive sampling with the aim of obtaining a sample representative of asthma management within each participating site.

The objectives of this study were to describe the demographics and clinical features of the asthma population by asthma severity and to estimate both SABA and ICS prescriptions per patient and within the different SABA and ICS groups. At each site, during a single study visit, prespecified patient data were collected by healthcare providers (HCPs) and collated into a centrally designed eCRF.

### Study population

Patients aged ≥ 12 years with a documented diagnosis of asthma, ≥ 3 consultations with the HCP or practice and medical records containing data for ≥ 12 months prior to the study visit were included. Patients with a diagnosis of other chronic diseases such as chronic obstructive pulmonary disease (COPD) or an acute or chronic condition that, in the opinion of the investigator, would limit their ability to participate in the study were excluded from the study. Signed informed consent was collected from participating patients or legal guardians of patients aged < 18 years.

### Variables and outcomes

As previously described [18], patients were categorised by their SABA and ICS prescriptions in the 12 months prior to the study visit. SABA prescriptions were categorised as 0, 1–2, 3–5, 6–9, 10–12 and ≥ 13, with over-prescription defined as ≥ 3 SABA canister prescriptions per year [12, 19, 20]. ICS canister prescriptions during the previous 12 months were recorded and categorised according to the prescribed average daily dose as low, medium or high [21].

Other variables included sociodemographic characteristics (age, number of comorbid conditions, body mass index [BMI], smoking status, educational level [primary or secondary school, high school or university and/or post-graduate education], medication reimbursement status [not reimbursed, partially reimbursed or fully reimbursed] and practice type [primary or specialist care]). Patients were also characterised based on investigator-classified asthma severity, guided by GINA 2017 treatment steps (GINA steps 1–2, mild asthma; GINA steps 3−5, moderate-to-severe asthma) [21]. The time since asthma diagnosis was recorded, together with the number of severe exacerbations in the preceding 12 months, which was based on the American Thoracic Society/European Respiratory Society recommendations, and defined as a worsening of asthma symptoms requiring hospitalisation, an emergency room visit or the need for intravenous corticosteroids or oral corticosteroids (OCS) for ≥ 3 days or a single intramuscular corticosteroid dose [22].

In addition, data on prescriptions for asthma medications, including ICS, fixed-dose combinations of ICS with long-acting β_2_-agonists (LABAs), OCS burst treatment, OCS maintenance treatment and antibiotics prescribed for asthma in the preceding 12 months were collected. Data on pharmacy purchases of over-the-counter (OTC) SABA without a prescription were also recorded. Asthma symptom control was evaluated using the GINA 2017 assessment of asthma control [21] and categorised as well controlled, partly controlled or uncontrolled.

### Statistical analysis

Descriptive analyses were used to characterise patients according to baseline demographics and clinical characteristics. Continuous variables were summarised as the number of non-missing values, mean, standard deviation (SD), median and range. Categorical variables were summarised as frequency counts and percentages.

## Results

### Patient disposition and clinical and sociodemographic characteristics

#### Patient disposition

**Fig. 1:**
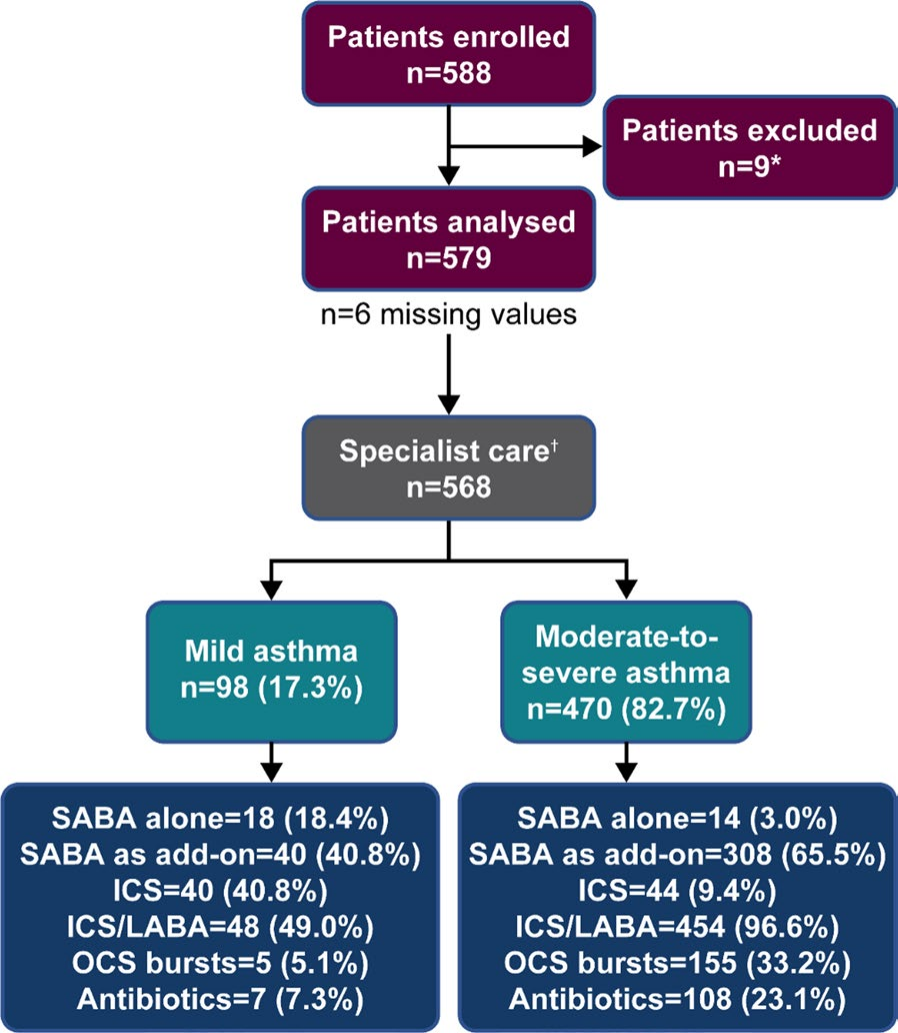
Patient disposition and study population by investigator-classified asthma severity in the SABINA III Turkey Cohort. *Patients excluded had a history of asthma < 12 months. ^†^Asthma severity data are reported for 568 patients and not for the six patients with missing data and the five patients erroneously categorised under primary care. ICS, inhaled corticosteroids; LABA, long-acting β_2_-agonist; OCS, oral corticosteroids; SABA, short-acting β_2_-agonist; SABINA, SABA use IN Asthma

Overall, 588 patients were enrolled in the study. However, nine patients were excluded due to an asthma duration of < 12 months. Subsequently, 579 patients were included in the overall analysis (Fig. 1). All patients were treated by specialists, including pulmonologists/respiratory physicians, immunologists, allergists and paediatricians. However, five patients were erroneously allocated to ‘Primary Care’, and six patients had missing values for ‘Practice Type’. Therefore, data on overall disease characteristics and treatment patterns are reported for 579 patients, whereas data on asthma severity (‘mild’ versus ‘moderate-to-severe’) are reported for 568 patients.

#### Patient characteristics

The mean (SD) age of patients was 47.4 (16.1) years, with more than half of patients (57.2%) aged 18–54 years. The majority of patients were female (74.3%) and had never smoked (68.6%; Table 1). The mean (SD) BMI of patients was 28.4 (5.9) kg/m^2^ and as per the World Health Organization classification criteria [23], 26.6% of patients had normal BMI, 35.1% were overweight and 35.4% were obese. Overall, just over half of patients had received high school education (25.4%) or obtained university and/or post-graduate education (26.4%). In line with the system of Universal Health Insurance (*Genel Sağlık Sigortası*) in Turkey [24–26], almost all patients (97.1%) reported fully reimbursed healthcare.

**Table 1:** Sociodemographic and clinical characteristics according to investigator-classified asthma severity in the SABINA III Turkey Cohort

	All (N = 579)	Patients classified by asthma severity (n = 568)*	
		Investigator-classified mild asthma (n = 98)	Investigator-classified moderate-to-severe asthma (n = 470)
**Age (years)**			
Mean (SD)	47.4 (16.1)	40.7 (19.2)	48.8 (15.0)
**Age group (years), n (%)**			
12–17	32 (5.5)	13 (13.3)	18 (3.8)
≥ 18–54	331 (57.2)	58 (59.2)	268 (57.0)
≥ 55	216 (37.3)	27 (27.6)	184 (39.1)
**Sex, n (%)**			
Female	430 (74.3)	61 (62.2)	362 (77.0)
**BMI (kg/m**^**2**^**)**^†^			
Mean (SD)	28.4 (5.9)	26.4 (5.1)	28.7 (6.0)
**BMI groups (kg/m**^**2**^**)**^†^**, n (%)**			
< 18.5	17 (2.9)	7 (7.1)	10 (2.1)
18.5–24.9	154 (26.6)	26 (26.5)	128 (27.2)
25–29.9	203 (35.1)	46 (46.9)	151 (32.1)
≥ 30	205 (35.4)	19 (19.4)	181 (38.5)
**Education level, n (%)**			
Primary and/or secondary school	266 (46.0)	37 (37.8)	222 (47.2)
High school	147 (25.4)	28 (28.6)	116 (24.7)
University and/or post-graduate education	153 (26.4)	33 (33.7)	119 (25.3)
Not established	13 (2.2)	0 (0.0)	13 (2.8)
**Healthcare insurance/medication funding, n (%)**			
Not reimbursed	0 (0.0)	0 (0.0)	0 (0.0)
Partially reimbursed	11 (1.9)	2 (2.0)	9 (1.9)
Fully reimbursed	562 (97.1)	95 (96.9)	456 (97.0)
Not established	6 (1.0)	1 (1.0)	5 (1.1)
**Smoking status history, n (%)**			
Active smoker	64 (11.1)	9 (9.2)	54 (11.5)
Former smoker	118 (20.4)	19 (19.4)	97 (20.6)
Never smoker	397 (68.6)	70 (71.4)	319 (67.9)

BMI, body mass index; SABINA, SABA use IN Asthma; SD, standard deviation; WHO, World Health Organization

*Asthma severity data are reported for 568 patients and not for the six patients with missing data or the five patients erroneously categorised under primary care

^†^BMI categorisation is based on WHO classification [23]

#### Disease characteristics

Patients had a median (minimum, maximum) asthma duration of 9.0 (1.0, 56.0) years (Table 2). Most patients (82.7%) had investigator-classified moderate-to-severe asthma (GINA treatment steps 3‒5), whereas 17.3% had mild asthma (GINA treatment steps 1‒2). Most patients were at GINA treatment step 4 (32.3%) or step 5 (32.0%). Patients reported a mean (SD) of 1.2 (1.8) severe asthma exacerbations and 46.5% experienced ≥ 1 severe asthma exacerbation in the 12 months preceding study initiation. A greater proportion of patients with moderate-to-severe asthma experienced ≥ 1 severe exacerbation in the previous 12 months compared with those with mild asthma (52.6% vs 17.3%). Overall, 67.0% of patients had ≥ 1 comorbidity; a higher percentage of patients with moderate-to-severe asthma had ≥ 1 comorbidity compared with those with mild asthma (69.8% vs 52.0%). The level of asthma symptom control was assessed as well controlled in 43.7%, partly controlled in 32.5% and uncontrolled in 23.8% of patients. Compared with patients with moderate-to-severe asthma, a higher proportion of patients with mild asthma had well-controlled asthma (67.3% vs 38.7%).

**Table 2:** Asthma characteristics according to investigator-classified asthma severity in the SABINA III Turkey Cohort

	All (N = 579)	Patients classified by asthma severity (n = 568)*	
		Investigator-classified mild asthma (n = 98)	Investigator-classified moderate-to-severe asthma (n = 470)
**Asthma duration (years)**			
Mean (SD)	12.0 (9.9)	8.6 (7.7)	12.6 (10.2)
Median (min, max)	9.0 (1.0, 56.0)	6.0 (1.0, 40.0)	10.0 (1.0, 56.0)
**Number of severe asthma exacerbations 12 months before the study visit**			
Mean (SD)	1.2 (1.8)	0.3 (0.8)	1.3 (1.9)
**Number of severe asthma exacerbations 12 months before the study visit by group, n (%)**			
0	310 (53.5)	81 (82.7)	223 (47.4)
1	105 (18.1)	8 (8.2)	96 (20.4)
2	61 (10.5)	6 (6.1)	53 (11.3)
3	48 (8.3)	2 (2.0)	46 (9.8)
> 3	55 (9.5)	1 (1.0)	52 (11.1)
**GINA classification, n (%)**			
Step 1	39 (6.7)	39 (39.8)	0 (0.0)
Step 2	60 (10.4)	59 (60.2)	0 (0.0)
Step 3	108 (18.7)	0 (0.0)	105 (22.3)
Step 4	187 (32.3)	0 (0.0)	184 (39.1)
Step 5	185 (32.0)	0 (0.0)	181 (38.5)
**Level of asthma symptom control, n (%)**			
Well controlled	253 (43.7)	66 (67.3)	182 (38.7)
Partly controlled	188 (32.5)	25 (25.5)	159 (33.8)
Uncontrolled	138 (23.8)	7 (7.1)	129 (27.4)
**Number of comorbidities, n (%)**			
None	191 (33.0)	47 (48.0)	142 (30.2)
1–2	300 (51.8)	36 (36.7)	258 (54.9)
3–4	78 (13.5)	14 (14.3)	61 (13.0)
≥ 5	10 (1.7)	1 (1.0)	9 (1.9)

GINA, Global Initiative for Asthma; max, maximum; min, minimum; SABINA, SABA use IN Asthma; SD, standard deviation

*Asthma severity data are reported for 568 patients and not for the six patients with missing data or the five patients erroneously categorised under primary care

### Prescribed asthma treatment

**Fig. 2:**
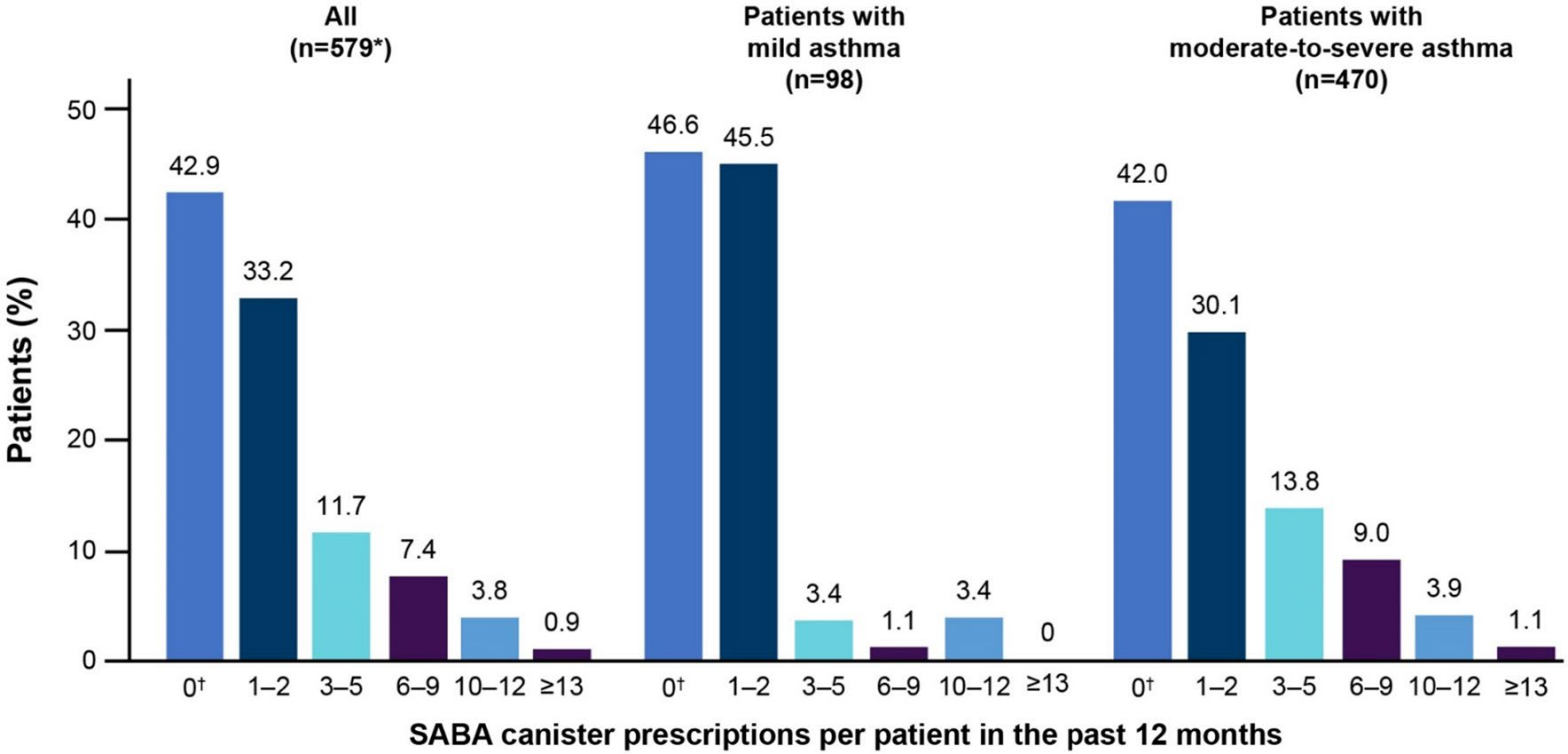
SABA prescriptions by investigator-classified asthma severity in the 12 months before the study visit in the SABINA III Turkey Cohort. *Asthma severity data are reported for 568 patients and not for the six patients with missing data or the five patients erroneously categorised under primary care. ^†^The category of patients classified as having 0 SABA canister prescriptions included patients using non-SABA relievers, non-inhaler forms of SABA and/or SABA purchased OTC. Missing data for the overall population: n = 136; mild asthma: n = 11; moderate-to-severe asthma: n = 125. OTC, over the counter; SABA, short-acting β_2_-agonist; SABINA, SABA use IN Asthma

Among patients prescribed SABA monotherapy or SABA in addition to maintenance therapy, 23.9% were prescribed ≥ 3 SABA canisters and 4.7% were prescribed ≥ 10 SABA canisters in the 12 months prior to study entry; 42.9% of patients were not prescribed any SABA (Fig. 2). A higher proportion of patients with moderate-to-severe asthma were prescribed both ≥ 3 (27.9%) and ≥ 10 SABA canisters (5.1%) than those with mild asthma (8.0% and 3.4%, respectively) in the previous 12 months.

#### SABA monotherapy

Overall, 5.7% of patients were prescribed SABA monotherapy, with a median (minimum, maximum) of 2.0 (1.0, 12.0) canisters in the previous 12 months. Of these, 35.5% of patients were prescribed ≥ 3 SABA canisters and 16.1% were prescribed ≥ 10 SABA canisters in the preceding 12 months (Table 3).

**Table 3:** SABA prescriptions in the 12 months before the study visit in the SABINA III Turkey Cohort

	All (N = 579)	Patients classified by asthma severity (n = 568)*	
		Investigator-classified mild asthma (n = 98)	Investigator-classified moderate-to-severe asthma (n = 470)
**Patients prescribed SABA monotherapy, n (%)**			
Yes	33 (5.7)	18 (18.4)	14 (3.0)
No	546 (94.3)	80 (81.6)	456 (97.0)
**Number of SABA canisters or inhalers per patient prescribed 12 months before the study visit**			
Number of patients	31	17	13
Mean (SD)	4.2 (3.9)	2.7 (3.5)	5.9 (3.8)
Median (min, max)	2.0 (1.0, 12.0)	2.0 (1.0, 12.0)	6.0 (2.0, 12.0)
**Number of SABA canisters or inhalers (as categories) per patient prescribed 12 months before the study visit, n (%)**			
0–2	20 (64.5)	15 (88.2)	5 (38.5)
3–5	0 (0.0)	0 (0.0)	0 (0.0)
6–9	6 (19.4)	0 (0.0)	5 (38.5)
10–12	5 (16.1)	2 (11.8)	3 (23.1)
≥ 13	0 (0.0)	0 (0.0)	0 (0.0)
Number of missing values	2	1	1
Total	31	17	13
**Patients prescribed SABA in addition to maintenance therapy, n (%)**			
Yes	356 (61.5)	40 (40.8)	308 (65.5)
No	223 (38.5)	58 (59.2)	162 (34.5)
**Number of SABA canisters or inhalers per patient prescribed 12 months before the study visit**			
Number of patients	222	30	189
Mean (SD)	3.5 (3.2)	2.2 (2.3)	3.7 (3.3)
Median (min, max)	2.0 (1.0, 16.0)	2.0 (1.0, 12.0)	2.0 (1.0, 16.0)
**Number of SABA canisters or inhalers per patient prescribed 12 months before the study visit in categories, n (%)**			
0–2	127 (57.2)	25 (83.3)	101 (53.4)
3–5	52 (23.4)	3 (10.0)	48 (25.4)
6–9	27 (12.2)	1 (3.3)	25 (13.2)
10–12	12 (5.4)	1 (3.3)	11 (5.8)
≥ 13	4 (1.8)	0 (0.0)	4 (2.1)
Number of missing values	134	10	119
Total	222	30	189

Max, maximum; min, minimum; SABA, short-acting β_2_-agonist; SABINA, SABA use IN Asthma; SD, standard deviation

*Asthma severity data are reported for 568 patients and not for the six patients with missing data or the five patients erroneously categorised under primary care

#### SABA in addition to maintenance therapy

Overall, 61.5% of patients were prescribed SABA in addition to their maintenance therapy, with a mean (SD) of 3.5 (3.2) canisters in the previous 12 months. Among the 222 patients for whom data were available, 42.8% were prescribed ≥ 3 SABA canisters and 7.2% were prescribed ≥ 10 SABA canisters in the preceding 12 months (Table 3). A higher proportion of patients with moderate-to-severe asthma were prescribed SABA in addition to maintenance therapy compared with those with mild asthma (65.5% vs 40.8%).

#### SABA purchase

Overall, 10.2% of patients purchased SABA OTC, of whom 27.1% purchased ≥ 3 SABA canisters in the previous 12 months (Table 4). A higher proportion of patients with moderate-to-severe asthma than those with mild asthma purchased ≥ 3 SABA canisters OTC without a prescription (29.4% vs 12.5%).

**Table 4:** Patients receiving SABA without a prescription in the 12 months before the study visit in the SABINA III Turkey Cohort

	All (N = 579)	Patients classified by asthma severity (n = 568)*	
		Investigator-classified mild asthma (n = 98)	Investigator-classified moderate-to-severe asthma (n = 470)
**Number of patients who received SABA without a prescription 12 months before the study visit, n (%)**			
Yes	59 (10.2)	8 (8.2)	51 (10.9)
No	514 (88.8)	90 (91.8)	413 (87.9)
Unknown	6 (1.0)	0 (0.0)	6 (1.3)
**Number of SABA canisters or inhalers per patient obtained without a prescription in categories, n (%)**			
1–2	43 (72.9)	7 (87.5)	36 (70.6)
3–5	15 (25.4)	1 (12.5)	14 (27.5)
6–9	0 (0.0)	0 (0.0)	0 (0.0)
10–12	1 (1.7)	0 (0.0)	1 (2.0)
≥ 13	0 (0.0)	0 (0.0)	0 (0.0)
Not applicable^✝^	0 (0.0)	0 (0.0)	0 (0.0)

eCRF, electronic case report form; SABA, short-acting β_2_-agonist; SABINA, SABA use IN Asthma

*Asthma severity data are reported for 568 patients and not for the six patients with missing data or the five patients erroneously categorised under primary care

^✝^‘Not applicable’ could be selected in the eCRF when patients purchased non-canister forms of SABA (e.g. oral or nebulised SABA) without a prescription

#### Asthma maintenance medications

Overall, 14.5% of patients were prescribed ICS, with a mean (SD) of 2.2 (2.0) canisters in the preceding 12 months. Of these patients, 52.4% were prescribed low-dose ICS, 30.5% medium-dose ICS and 17.1% high-dose ICS (Table 5). As expected, a higher proportion of patients with mild asthma (40.8%) received ICS compared with those with moderate-to-severe asthma (9.4%).

**Table 5:** Other asthma treatments prescribed in the 12 months before the study visit in the SABINA III Turkey Cohort

	All (N = 579)	Patients classified by asthma severity (n = 568)*	
		Investigator-classified mild asthma (n = 98)	Investigator-classified moderate-to-severe asthma (n = 470)
**Patients prescribed ICS, n (%)**			
Yes	84 (14.5)	40 (40.8)	44 (9.4)
No	495 (85.5)	58 (59.2)	426 (90.6)
**Total prescribed daily ICS dose, n (%)**			
Low dose	43 (52.4)	24 (63.2)	19 (43.2)
Medium dose	25 (30.5)	12 (31.6)	13 (29.5)
High dose	14 (17.1)	2 (5.3)	12 (27.3)
Number of missing values	2	2	0
Total	82	38	44
**Number of ICS canisters or inhalers per patient prescribed 12 months before the study visit**			
Number of patients	84	40	44
Mean (SD)	2.2 (2.0)	2.4 (2.1)	2.0 (1.9)
**Patients prescribed ICS/LABA (fixed-dose combination), n (%)**			
Yes	511 (88.3)	48 (49.0)	454 (96.6)
No	68 (11.7)	50 (51.0)	16 (3.4)
**Total prescribed daily ICS dose, n (%)**			
Low dose	97 (19.1)	28 (59.6)	67 (14.8)
Medium dose	244 (47.9)	15 (31.9)	226 (49.9)
High dose	168 (33.0)	4 (8.5)	160 (35.3)
Number of missing values	2	1	1
Total	509	47	453
**Patients prescribed OCS burst/short course, n (%)**			
Yes	164 (28.5)	5 (5.1)	155 (33.2)
No	412 (71.5)	93 (94.9)	312 (66.8)
Number of missing values	3	0	3
Total	576	98	467
**Patients prescribed OCS maintenance treatment, n (%)**			
Yes	18 (3.1)	0 (0.0)	17 (3.6)
No	561 (96.9)	98 (100.0)	453 (96.4)
**Patients prescribed antibiotics for asthma, n (%)**			
Yes	118 (20.5)	7 (7.3)	108 (23.1)
No	457 (79.5)	89 (92.7)	360 (76.9)
Number of missing values	4	2	2
Total	575	96	468

ICS, inhaled corticosteroids; LABA, long-acting β_2_-agonist; OCS, oral corticosteroids; SABINA, SABA use IN Asthma; SD, standard deviation

*Asthma severity data are reported for 568 patients and not for the six patients with missing data or the five patients erroneously categorised under primary care

ICS/LABA fixed-dose combination as maintenance therapy was prescribed to 88.3% of patients. Of these patients, 19.1% were prescribed low-dose ICS, 47.9% medium-dose ICS and 33.0% high-dose ICS (Table 5). A greater proportion of patients with moderate-to-severe asthma were prescribed an ICS/LABA fixed-dose combination compared with those with mild asthma (96.6% vs 49.0%). In patients with mild asthma who received an ICS/LABA fixed-dose combination, 70.8% were prescribed a formoterol-containing ICS/LABA (38.2% of whom received beclometasone/formoterol and 61.8% budesonide/formoterol; Additional file 1).

#### Oral corticosteroids

In the 12 months prior to study entry, OCS burst treatment was prescribed to 28.5% of patients, with a higher percentage of patients with moderate-to-severe asthma prescribed an OCS burst compared with those with mild asthma (33.2% vs 5.1%; Table 5).

#### Antibiotics

Overall, 20.5% of patients were prescribed antibiotics for asthma (Table 5). Prescriptions of antibiotics were higher in patients with moderate-to-severe asthma than in those with mild asthma (23.1% vs 7.3%).

Overall, 42% of patients were prescribed leukotriene receptor antagonists and 10.9% of patients were prescribed monoclonal antibodies in the 12 months before the study visit. Additionally, long-acting muscarinic antagonists, xanthines and short-acting muscarinic antagonists were prescribed to 7.9%, 2.9% and 1.7% of patients, respectively.

### Asthma treatments and exacerbations

When patients were stratified by treatments prescribed in the previous 12 months, most patients who were prescribed an OCS burst experienced ≥ 1 severe exacerbation (93.3%), followed by those prescribed antibiotics (82.2%) and SABA in addition to maintenance treatment (60.1%).

## Discussion

This nationwide cross-sectional study conducted as part of the SABINA III study to characterise the asthma patient population and describe the extent of SABA prescriptions provides valuable insights into asthma treatment practices in Turkey. Notably, 23.9% of patients for whom data were available were prescribed SABA in excess of current treatment recommendations (≥ 3 SABA canisters/year). Moreover, the burden of asthma was high, with 46.5% of patients experiencing ≥ 1 severe exacerbation in the previous 12 months.

In general, the baseline sociodemographics and disease characteristics of patients from Turkey were consistent with those of the SABINA III cohort [18]. Reflecting the rapid increase in obesity in Turkey over the past 20 years [27], 35.1% of patients were classified as overweight (BMI, 25–29.9 kg/m^2^) and 35.4% as obese (BMI, ≥ 30 kg/m^2^). More than one-third of patients who had moderate-to-severe asthma were obese; however, this finding is not unexpected, given that obesity increases the prevalence of asthma and is associated with poorer outcomes [28–30]. Strikingly, and in keeping with the results from the SNAPSHOT study in five Middle Eastern countries, including Turkey [3], 67% of patients from this Turkish cohort had ≥ 1 comorbidity. This highlights the need for careful assessment of comorbidities as part of routine clinical care, particularly because the presence of asthma-related comorbidities has been shown to negatively impact asthma control [31]. Furthermore, since Turkey provides comprehensive health coverage to its citizens [24–26], almost all patients (97.1%) received fully reimbursed healthcare; this is in stark contrast to the overall SABINA III population, wherein only 47.2% of patients were fully reimbursed [18]. Although all study sites were intended to be representative of healthcare practices across the country, all patients in this Turkish cohort were treated by specialists, resulting in most patients being classified with moderate-to-severe asthma (82.7%). This was likely due to inherent challenges commonly encountered in conducting clinical trials at a primary care level [32] as well as those observed in conducting real-world studies [33]. Consequently, this cohort of patients from Turkey represents a ‘better case scenario’, with virtually all patients receiving fully reimbursed healthcare and all patients receiving treatment under specialist care.

Overall, only 5.7% of patients, most of whom were classified with mild asthma, were prescribed SABA monotherapy in the preceding 12 months; this was expected, given that only 6.7% of patients were diagnosed as GINA step 1. However, 61.5% of patients were prescribed SABA in addition to maintenance therapy, with SABA prescriptions more common in patients with moderate-to-severe asthma than in those with mild asthma. Moreover, 42.8% of patients were prescribed ≥ 3 SABA canisters with maintenance therapy in the previous 12 months, which is considered over-prescription; worryingly, 7.2% of patients were prescribed ≥ 10 SABA canisters. This is of concern since overall findings from SABINA III, which included 8351 patients across 24 countries, indicated an association between high SABA prescriptions and poor clinical outcomes, with prescriptions of 3–5, 6–9, 10–12 and ≥ 13 SABA per year (vs 1 − 2) associated with increasingly lower odds of controlled or partly controlled asthma and higher rates of severe exacerbations across treatment steps and clinical care settings [18]. Nevertheless, overall prescriptions of ≥ 3 SABA canisters 12 months prior were considerably lower in Turkey (23.9%) than in the overall SABINA III population (38.0%) [18]. One possible explanation for this finding is that all patients in Turkey were treated at secondary and tertiary centres by specialists, who are likely more familiar with current asthma treatment guidelines and have the expertise to provide optimal care for patients with asthma [34], whereas SABINA III included patients also treated under primary care [18]. Hence, SABA over-prescription may have been higher if primary care physicians, who may be less familiar with updated guideline treatment recommendations, had participated in this study. Moreover, results on SABA prescriptions in Turkey may have been impacted by the relatively large proportion of missing data; therefore, SABA over-prescription in Turkey is potentially higher than documented. The omission of SABA prescription data highlights the importance of maintaining accurate medical records since excess SABA inhalers should be identified as a signal for poorly controlled asthma [35]. Taken together, these findings clearly indicate that there is a considerable proportion of patients with asthma across Turkey who are currently not optimally treated according to current GINA recommendations despite being under specialist care.

Importantly, not all SABAs were obtained with prescriptions. In fact, 10.2% of patients purchased SABA OTC in the previous 12 months, with over a quarter of patients (27.1%) purchasing ≥ 3 SABA canisters. This highlights patients’ over-reliance on SABA therapy and their willingness to self-manage worsening asthma symptoms [36–39]. This is particularly concerning because many patients who purchased SABA OTC likely did so in addition to their SABA prescriptions. However, this finding is not unexpected as it is common for patients to purchase medications OTC in Turkey. Indeed, results from a national survey of community pharmacists on their role in asthma management in Turkey indicated that 91.0% of pharmacists provided rescue medication without a prescription [40]. Unsurprisingly, therefore, recent studies in Turkey have reported that although pharmacists play a vital role in asthma care in Turkey, further education and training are required to improve both their knowledge of asthma and asthma control in Turkey [40, 41]. Similarly, there is a need to educate patients on self-management of asthma and drive policy changes to regulate SABA purchase without prescriptions, particularly because SABA purchase is associated with low rates of consultation with family practitioners and specialists, low use of prescription-only medication (particularly ICS) and undertreatment of asthma [39, 42, 43].

The majority of patients were prescribed either ICS or ICS/LABA fixed-dose combinations as maintenance medication. Overall, 14.5% of patients were prescribed ICS, which was in alignment with the fact that only 10.4% of patients were diagnosed as GINA step 2. However, a mean of only 2.2 canisters was prescribed in the previous 12 months, suggesting potential ICS underuse as one canister per month is considered good clinical practice. This finding is also a matter of concern because together with over-prescription of SABAs, insufficient provision of ICS-containing treatments has been identified as a preventable cause of death from asthma [35]. Interestingly, almost half of all patients with mild asthma were prescribed an ICS/LABA fixed-dose combination, which was not in alignment with GINA recommendations at the time this study was conducted [44, 45]. This observation may be indicative of prescription behaviour and the fact that physicians may be more comfortable prescribing an ICS/LABA fixed-dose combination instead of ICS to maximise patient adherence and ensure optimal symptom control. Moreover, some physicians may be reluctant to step down asthma treatment, even in patients with well-controlled asthma. However, this finding aligns with the results of a recent evaluation of inhaler therapies in respiratory diseases conducted in Turkey between 1998 and 2015, which reported that following the introduction of ICS/LABA fixed-dose combinations in 2002, their use has increased and they have become the most commonly used treatments in Turkey [46]. Notably, although over 80% of patients in this Turkish cohort were classified with moderate-to-severe asthma, only 10.9% of patients were prescribed a monoclonal antibody, a finding likely attributable to their high cost [8, 47]. However, a high proportion of patients (over 40%) were prescribed a LTRA, which may be due to their ease of administration and reassuring safety profile [48]. Alternatively, patients may have been prescribed a LTRA to treat common asthma comorbidities such as allergic rhinitis [49] or nasal polyps [50] or for better asthma control in patients with predominantly moderate-to-severe asthma [51].

More than a quarter of patients (28.5%), predominantly those with moderate-to-severe asthma, were prescribed OCS burst treatment. This was presumably to treat exacerbations because 93.3% of patients who were prescribed OCS burst treatment experienced ≥ 1 severe exacerbation, further emphasizing that patients in this Turkish cohort had suboptimal asthma control. However, this high percentage of patients prescribed OCS burst treatment may also reflect the fact that physicians often prescribe OCS as rescue medication in the event of worsening asthma symptoms or as part of a written asthma action plan. Furthermore, despite the introduction of two main antimicrobial stewardship programmes in Turkey [52], and a significant reduction in overall antibiotic prescriptions between 2011−2018 following governmental interventions at a national level [53], 20.5% of patients were prescribed antibiotics for asthma, indicating a lack of familiarity with asthma guidelines that do not support the routine use of antibiotics unless there is strong evidence of a lung infection [45]. Indeed, results from a Cochrane Database Systematic Review found limited evidence that antibiotics administered at the time of an asthma exacerbation may improve symptoms and peak expiratory flow rate at follow-up compared with standard care or placebo [54]. In addition, results from a real-life comparative effectiveness study concluded that the routine addition of antibiotics to OCS in the management of asthma exacerbations provided little clinical benefit [55].

Notably, even though all patients were treated by specialists and the majority reported full healthcare reimbursement, less than half of patients (43.7%) had well-controlled asthma and 23.8% of patients had uncontrolled asthma. These findings are aligned with those from the SABINA III cohort, where 17.2% of patients were recruited by primary care physicians, and just 43.2% of patients had well-controlled asthma [18] Likewise, results from SABINA Turkey are consistent with those from a cross-sectional multicentre survey in 2,336 patients with asthma recruited from seven geographical districts in Turkey, which reported that only 51.5% of patients had controlled asthma, as assessed by the Turkish version of the Asthma Control Test [56]. Consequently, the burden of asthma in SABINA Turkey was high, with 46.5% of patients experiencing at least one severe asthma exacerbation in the previous 12 months. Even though a greater proportion of patients with moderate-to-severe asthma experienced ≥ 1 severe asthma exacerbation in the previous year, 17.3% of patients with mild asthma also reported ≥ 1 severe asthma exacerbation, suggesting the potential underestimation of patients with milder disease or the inappropriate management of patients with mild asthma, resulting in poor symptom control. Taken together, these findings highlight the need for educational initiatives targeting both patients and physicians to optimise asthma care. To this end, it is anticipated that the Global Alliance against Chronic Respiratory Diseases (GARD) Turkey project, a voluntary alliance launched by the Turkish Ministry of Health on chronic airway disease (asthma and COPD), will improve asthma outcomes through the creation of working groups focussed on monitoring chronic respiratory diseases, advocacy and awareness of the programme, disease prevention as well as early detection and effective treatment and prevention of complications, including educating patients and HCPs about appropriate treatment [57].

Results from this study should be interpreted in light of several limitations. Firstly, this study used purposive sampling that can be highly prone to research bias. SABA prescriptions were used as a proxy for SABA use; thus, SABA prescriptions may not reflect actual SABA use or treatment adherence. In addition, because data entry into the eCRF relied on the physician’s assessment, findings may have been impacted by misinterpretation of instructions and recall bias. All patients were recruited from specialist care, leading to a greater number of patients with moderate-to-severe asthma being recruited into the study. Thus, the study population may not be truly representative of the overall asthma patient population or reflect the way asthma is currently being managed in Turkey. Consequently, additional studies are required at the primary care level to gain a more comprehensive understanding of treatment practices across Turkey. Moreover, data on the use of alternative relievers such as ICS/formoterol combinations was not recorded. Similarly, the percentage of patients prescribed a maintenance and reliever therapy regimen was also not captured. Finally, the large amount of missing data on SABA prescriptions in the 12 months before the study visit precluded a thorough and accurate assessment of the extent of SABA use in Turkey.

In summary, to the best of our knowledge, this is the first study specifically designed to examine the extent of SABA prescriptions in Turkey. Crucially, the use of a standardised threshold for defining SABA over-prescription enabled an assessment of SABA use not only across Turkey but also globally across regions and countries, whilst the centralised eCRF can be a source of public health data. The collection of these real-world data on SABA over-prescription will enable policymakers and clinicians to make targeted changes in clinical practices to improve asthma outcomes in Turkey.

## Conclusion

Despite treatment under specialist care and fully reimbursed healthcare, results from the Turkish cohort of the SABINA III study indicated SABA over-prescription (≥ 3 canisters in the previous 12 months) in nearly a quarter of all patients. Furthermore, 10.2% of patients purchased SABA OTC without a prescription. Overall, less than half of all patients had well-controlled asthma (43.7%) and 46.5% of patients experienced ≥ 1 severe asthma exacerbation in the preceding year. These findings highlight that SABA over-prescription is a public health issue in Turkey, necessitating the need for HCPs and policymakers to work together to promote educational initiatives and ensure that clinical practices align with the latest evidence-based recommendations.

## Supplementary Information

**Additional file 1.** Maintenance medication categorised by asthma severity in the 12 months before the study visit in the SABINA III Turkey Cohort.

## Data Availability

The data that support the findings of this study are available from AstraZeneca but restrictions apply to the availability of these data (https://astrazenecagrouptrials.pharmacm.com/ST/Submission/Disclosure), which were used under license for the current study, and so are not publicly available. Data are however available from Dr. Arzu Yorgancıoğlu upon reasonable request and with permission of AstraZeneca.

## Abbreviations

AIRET: Asthma Insights and Reality in Turkey
BMI: Body mass index
COPD: Chronic obstructive pulmonary disease
eCRF: Electronic case report form
GARD: Global alliance against chronic respiratory diseases
GINA: Global initiative for asthma
HCP: Healthcare provider
ICS: Inhaled corticosteroid
LABA: Long-acting β-agonist
OCS: Oral corticosteroids
OTC: Over the counter
SABA: Short-acting β_2_-agonist
SABINA: SABA use IN Asthma
SD: Standard deviation
