# Supplementary material for: Short-acting β2-agonist prescription patterns in patients with asthma in Turkey: results from SABINA III
Source: BMC Pulm Med. 2022 Jun 2;22:216. doi: 10.1186/s12890-022-02008-9 (PMC9161536; doi:10.1186/s12890-022-02008-9)
Supplement: Supplementary file 1 — Additional file 1. Maintenance medication categorised by asthma severity in the 12 months before the study visit in the SABINA III Turkey Cohort. [file 12890_2022_2008_MOESM1_ESM.docx]

**Additional File 1:** Maintenance medication categorised by asthma severity in the 12 months before the study visit in the SABINA III Turkey Cohort

| **GINA classification** | **Prescribed medication** | **ICS/LABA active substance** | **Total number of patients (n = 511)** |
| --- | --- | --- | --- |
| Step 1 | As needed SABA | Fluticasone/salmeterol | 3 |
|  |  | Beclometasone/formoterol | 4 |
|  |  | Budesonide/formoterol | 9 |
| Step 2 | Regular low-dose ICS +/- SABA | Fluticasone/ salmeterol | 9 |
|  |  | Beclometasone/formoterol | 9 |
|  |  | Budesonide/formoterol | 12 |
|  |  | Fluticasonefuroate/vilanterol | 2 |
| Step 3 | Low-dose ICS/LABA +/- SABA | Fluticasone/salmeterol | 24 |
|  |  | Beclometasone/formoterol | 26 |
|  |  | Budesonide/formoterol | 41 |
|  |  | Fluticasonefuroate/vilanterol | 9 |
| Step 4 | Medium dose ICS/LABA + add on maintenance medication +/- SABA | Fluticasone/ salmeterol | 49 |
|  |  | Beclometasone/formoterol | 39 |
|  |  | Budesonide/formoterol | 87 |
|  |  | Fluticasonefuroate/vilanterol | 7 |
| Step 5 | High dose ICS/LABA + add on maintenance medication +/- SABA | Fluticasone/salmeterol | 53 |
|  |  | Beclometasone/formoterol | 39 |
|  |  | Budesonide/formoterol | 71 |
|  |  | Fluticasonefuroate/vilanterol | 18 |

GINA, Global Initiative for Asthma; ICS, inhaled corticosteroids; LABA, long-acting β_2_-agonist; SABA, short-acting β_2_-agonist
